# News and Events of the Week

**Published:** 1910-08-06

**Authors:** 


### News and Events of the Week.

### BRITISH MEDICAL ASSOCIATION.

### SOME SECTIONAL POINTS.

Anatomy.—Professor Keith, in his presidential address, pointed out the necessity for concerted action on the part of the schools and teachers to consider the supply of material. The importance of the pharyngeal tonsil was discussed by Professor Symington, who holds that enlargement rarely interferes with nasal breathing or causes deafness, and that it rarely persists after puberty.

Dermatology.—The treatment of syphilis was discussed, but no new points were raised. Several speakers deprecated the manner in which students were taught the subject. Intra-muscular injections were well spoken of

Children.—Dr. Coutts, in an interesting paper on non-tubercular joint diseases, pointed out the anomalies in the present system of classification and discussed some points of treatment. Mr. Corner discussed the surgical aspects and narrated his unsatisfactory experience of the vaccine treatment in streptococcal cases. Mr. Murphy thought that gonococcal arthritis was relatively rare in children in England. Mr. Elmslie warned the meeting against accepting cases of painless joint tubercle as rheumatic in origin, and could not accept the statement that the joint lesions met in polio-myelitis were cases of Charcot's joints. Mr. Wynter had found camphor valuable in the treatment of pneumococcal arthritis. Following this discussion came Dr. Gauvain's interesting and valuable paper on the conservative treatment of tuberculous cripples as carried out at the Home at Alton.

Throat and Ear.—Dr. von Eickens's paper on the direct examination of the air passages was mainly a description of modern technical methods. He prefers chloroform to ether, sometimes using a local anæsthetic at the same time. In the section on Otology an interesting discussion on aural tuberculosis in children was the feature of the day. The incidence of aural tubercle is very low, and several speakers commented upon its extreme rarity. The general consensus of opinion was in favour of surgical treatment. Dr. Dundas Grant advocated the use of methyl violet as an antiseptic in such cases.

Physiology.—A discussion on food values. The general opinion was against excessively low standards of diet as advocated by Chittenden.

Psychology.—Dr. Savage, dealing with marriage and insanity, referred to the general opinion that the marriage of all who had been insane was to be opposed, but thought that the special form of insanity should be taken into consideration before a general dictum was expressed. Personally he would debar from marriage all persons who had suffered from delusional insanity, those who had suffered from epileptic attacks associated with mental disorder, and those who had a well-marked history of periodical attacks. He was also opposed to the marriage of diabetics or syphilitics. An interesting discussion followed, the speakers generally voicing the opinion that, however desirable any limitation of marriage in those circumstances might be, the time was not yet ripe for legislation on the subject.

State Medicine.—In this section an interesting discussion was opened by Mrs. Webb, who dealt with the broad aspects of public health administration. We hope to deal with this discussion on some future occasion in detail.

Surgery.—The section on Surgery occupied itself with a debate on the question of the operative treatment of simple fractures, which was introduced by Mr. Lane, whose views found considerable support. Mr. Fullerton spoke on behalf of the conservative treatment, and Professor Willems also laid stress on the importance of more thorough attention to the employment of apparatus and thus avoiding, where possible, the open operation.

Pharmacology.—The sour milk treatment was fully discussed. Professor Hewlett stated that tablets were not good for preparing efficient lactic acid preparations. Dr. Bryce pointed out some of the evil results that had been known to result from indiscriminate use of sour milk. One of these was the production of constipation. Professor Sahli had found the treatment of some use in acidosis and in enteric, and thought that the good results were mainly due to the lactic acid.

In the section on Pathology an animated discussion followed the reading of Dr. Mott's paper on chronic alcoholism. In the section on Ophthalmology two papers were read dealing with lesser-known methods of treatment—Messrs. Lawson and Davidson's paper on the treatment of certain eye conditions with radium, and Dr. Traquair's communication on zinc ionisation in cases of purulent keratitis.

In the Medical section the discussion was on the question of acidosis, and stress was laid by Professor Edsall, who initiated the discussion, on the use of alcohol in the treatment of cases of acidosis. He uses the drug in comparatively large doses, and has also found glycerine-aldehyde of service. Dr. E. Spriggs advocated prophylactic treatment; the only palliative treatment was by means of sodium bicarbonate.

Diseases of Women.—Dr. Herman opened the discussion on dysmenorrhœa, and stated that the sole natural cure was pregnancy. For relieving pain morphia might be cautiously used; phenacetin was less harmful but often of little use; but guaiacum was perhaps the most generally effective drug to use. Gradual dilatation, and finally ovariotomy or hysterectomy might have to be considered in obstinate cases. Subsequent speakers referred to other forms of treatment which had been found of use: alcoholic abdominal compresses as hot as the patient could bear, electrical treatment, the Fleiss method, and Dudley's operation. Puerperal fever was the subject of an interesting paper by Professor Döderlein, in which he advocated the routine use of gloves in making vaginal examinations on lying-in women.

---

His Majesty the King has graciously consented to become Patron of the British Medical Association.

---

A medical branch of the Navy League has been formed. The branch has already been practically in existence, although the meetings so far have only been those of the small provisional committee. Sir R. Douglas Powell is the president, Dr. Maurice Craig hon. secretary, and Dr. Percy Smith hon. treasurer.

---

The death is announced of Mr. Lawrence Holden, of Lancaster, who for over half a century was coroner for the Hundreds of North and South Lonsdale (except the Liberty of Furness), and was probably the oldest coroner in England. Many years ago he committed for trial two attendants at the Lancaster Asylum who were accused of causing the death of a patient by ill-treatment, admitting the evidence of an asylum patient, and his ruling was upheld by the High Court Judge.

### Lord Mayor Treloar's Home and College.

On Saturday last about two hundred members of the British Medical Association accepted the invitation of Sir William Treloar to visit the Cripples Home and College at Alton, in Hampshire. The party travelled by special train, and were received on arrival by Sir William Treloar, Sir Ernest Flower, and Sir W. Dunn, M.P., the trustees.

After visiting the various wards, the nurses' home, and the college, luncheon was served in the recreation room, Sir William Treloar explained the circumstances under which the home and college had been established, and the objects which it was promoted to serve.

Like many other national institutions, it has grown from very small beginnings, and it takes its origin from an effort made nearly twenty years ago to brighten the homes of crippled children at Christmas time by the gift of a hamper of good things. "It was in this way," said Sir William, "that I became acquainted with the large number of crippled children in London, and understood how great an army of little sufferers the Metropolis alone contained. When I became Lord Mayor I felt that I had an opportunity to do something more than a passing kindness, and to appeal from the great platform of the Mansion House to the public for help to establish a national home and college for young cripples.

"The children come from all parts of the country to be treated under conditions which are not possible for them to obtain in any of the great hospitals of the country. I need not say that the treatment of children suffering from tuberculous disease of the bones is, of necessity, a long and costly process, and the demand on the accommodation of our hospitals is such that in most cases these children have to be treated as out-patient., since the beds in our hospitals must be devoted to acute cases, and cannot be allotted to the tuberculous cripple until the time comes when the disease is so threatening that instant active treatment is necessary.

"The object which we have is the curative treatment of such cases. Here in this beautiful country home, amidst pure air and wholesome surroundings, the children stay with us till they are cured, or, at the worst, until every effort has been made to combat successfully the disease. The general treatment consists of the open-air life, with abundant plain wholesome food, and such general tonics as may from time to time be required. In the treatment of most of the cases time is essential. If the cure is to be complete the stay in the home must be a protracted one. We have, therefore, arranged to give to the children such elementary education as they are capable of receiving. The education which we provide is of a simple but useful kind. The children are classed and arranged in groups, so that the more forward child shall not be hampered by having to wait for the less intelligent. Some—and they are fairly numerous—who come to us quite ignorant are instructed in the rudiments of education. We teach them to read, write, and spell correctly, and the more simple rules of arithmetic, with some elementary instruction in English history and geography. In the summer the education of the children is given in the forest school."

Addresses were also given by the other trustees: Mr. D'Arcy Power, Mr. Pett Ridge, and Dr. Gouvain, the Resident Medical Officer, who gave a demonstration of a special method, which he had introduced for making plaster of Paris splints for spinal and hip cases.

The college boys were afterwards seen at physical drill, and the company returned to London by a special train later in the afternoon.

Some members of the B.M.A. took occasion of the cordial invitation extended by the Mayors of Brighton and Hove to visit these health-resorts on Saturday last. They were entertained at luncheon by the Mayor of Hove and the local members of the medical profession, and subsequently by the Mayor and Mayoress of Brighton to a garden party in the grounds of the Royal Pavilion.

A party of some two hundred members of the British Medical Association went to Bath on Saturday at the invitation of the Mayor and Town Council. The visitors were received by the Mayor and Mayoress and entertained to luncheon. The Mayor of Bath, in proposing the health of the Association, referred to the thermal bathing establishment and pointed out how it had progressed since the Association held its Conference in Bath in 1878. Provision had lately been made for drinking the hot mineral waters in the open air amid pleasant surroundings and to the accompaniment of music. Dr. Mott, replying for the visitors, alluded to the great development of Continental spas as illustrated by Vichy, the waters of which, however, he did not consider superior to those of Bath. They had heard at their meetings in London of the wonderful power of radium in the treatment of cancer, and radium existed in the Bath waters. English doctors should exhibit patriotism by sending patients to home instead of foreign spas. The visitors afterwards inspected the bathing establishments, and several remained over for the week-end to try for themselves the excellence of the arrangements.

H.M. the King has been pleased, by Warrant under his Majesty's Royal Sign Manual, bearing date the 20th inst., to appoint Dr. Hamilton Clelland Marr, M.D., to be a Commissioner in Lunacy for Scotland in the room of Dr. John Fraser, M.B., C.M., retired.

The returns of the Metropolitan Asylums Board for the two weeks ended July 23 show that three cases of smallpox have been dealt with at the Joyce Green Hospital, Darenth, Kent. One patient has since been discharged from hospital.

A luncheon was given at the Carlton Hotel on Friday the 28th ult. to the delegates of the Japanese Red Cross Society now in England. Mr. S. Hirayama, replying to the toast of the evening, said the Japanese Red Cross Society, like the British Red Cross Society, was favoured with the patronage and protection of the Imperial Royal Family; and it was not easy to estimate the impetus this gave to the rapid extension of the work of the Red Cross. It was now devoting all its energy to the development of a perfect system of relief which could be instantly put into operation in time of war.

